# Integrating Value Considerations in the Decision Making for the Design of Biorefineries

**DOI:** 10.1007/s11948-020-00251-z

**Published:** 2020-07-07

**Authors:** Mar Palmeros Parada, Lotte Asveld, Patricia Osseweijer, John Alexander Posada

**Affiliations:** Biotechnology Department, Delft University of Technology, Van der Maasweg 9, 2629HZ Delft, The Netherlands

**Keywords:** Biorefinery design, Bioplastics, Midstream modulation, Value sensitive design, Sustainable biorefineries

## Abstract

Biobased production has been promoted as a sustainable alternative to fossil resources. However, controversies over its impact on sustainability highlight societal concerns, value tensions and uncertainties that have not been taken into account during its development. In this work, the consideration of stakeholders’ values in a biorefinery design project is investigated. Value sensitive design (VSD) is a promising approach to the design of technologies with consideration of stakeholders’ values, however, it is not directly applicable for complex systems like biorefineries. Therefore, some elements of VSD, such as the identification of relevant values and their connection to a technology’s features, are brought into biorefinery design practice. Midstream modulation (MM), an approach to promoting the consideration of societal aspects during research and development activities, is applied to promote reflection and value considerations during the design decision making. As result, it is shown that MM interventions during the design process led to new design alternatives in support of stakeholders' values, and allowed to recognize and respond to emerging value tensions within the scope of the project. In this way, the present work shows a novel approach for the technical investigation of VSD, especially for biorefineries. Also, based on this work it is argued that not only reflection, but also flexibility and openness are important for the application of VSD in the context of biorefinery design.

**Electronic supplementary material:**

The online version of this article (10.1007/s11948-020-00251-z) contains supplementary material, which is available to authorized users.

## Introduction

It is now more than a decade since controversies over the sustainability of biofuels began to surface (Rosegrant and Msangi 2014). These controversies called attention to societal concerns, value tensions and uncertainties that had not been considered during the development of biobased production. For example, once biofuel production started to increase in the 2000s, its association with food production and land use change started to be debated (Rosegrant and Msangi 2014). As consequence, tensions emerged between these sustainability aspects and the emission reduction objectives that drove biofuel production in the first place. While these concerns do not necessarily relate to all biobased products, they do illustrate some of the complexities that can arise around this production approach.

Biorefineries are the processes and systems for the production of fuels, materials and chemicals from biobased resources (Bauer et al. 2017). During the design of biorefineries, the various alternatives that can define them are explored, including feedstock types, technological platforms, and by-products. Therefore, addressing stakeholder concerns about sustainability and acknowledging value tensions during the design of these systems can contribute to the development of more sustainable and acceptable biobased production. Several methods have been developed to consider sustainability during the design of biorefineries. However, these methods are typically closed to stakeholder participation and are often limited to issues that already drive biobased production, such as energy efficiency and the reduction of carbon emissions (Palmeros Parada et al. 2017; Pfau et al. 2014). This means that existing biorefinery design approaches rarely address societal concerns, value tensions and uncertainties related to the sustainability of biobased production.

Value sensitive design (VSD) is an approach to proactively designing in support of stakeholders’ values (Friedman et al. 2008a, b). However, there has been limited work done on the application of VSD for technological systems such as biorefineries, where diverse stakeholders across various geographies and sectors play a role. Additionally, at early stages of development of the biorefinery, there is limited availability of information, and involvement of stakeholders is difficult; at later stages the capacity to change the project is limited once investments have been made. An explorative VSD research on the investigation of stakeholders’ values and the generation of project specific principles for the early-stage design biorefineries has been published recently (Palmeros Parada et al. 2018). In their analysis, the authors suggest that these principles could guide subsequent design activities for obtaining a value sensitive biorefinery concept. However, there was no empirical work on the use of this analysis to derive a design concept (i.e. a biorefinery), and how to implement it as part of a design project.

In this work we investigate the development of an early-stage biorefinery design project by a design team. Specifically, the aim of this work is to explore how considerations of stakeholders’ values can be integrated to the decision making processes that lead to a biorefinery concept. Taking as starting point the work by Palmeros Parada et al. (2018), the hypothesis is that this integration can be achieved by promoting reflection during the design activities. To put this into practice, an approach to promoting the consideration of societal aspects during research and development (R&D) activities called midstream modulation (MM) (Fisher et al. 2006) is adapted to encourage a design team to reflect on identified stakeholders’ values. Therefore, while we argue that this work contributes to the application of VSD by bringing considerations of stakeholders’ values during the design of complex systems like biorefineries, the focus here is on the design process itself and not so much on the outcome (the biorefinery concept).

## Background

Prior to describing the methodology, the theories and concepts that serve as a basis for this work will be introduced in the following paragraphs.

### Biorefinery Design

Biorefineries are defined as the processes, facilities, and production systems for obtaining biobased products. In the broadest sense, biorefineries can be spread across various locations and include different stages of a product value chain (Bauer et al. 2017), such as the processing of agricultural residues and the conversion steps for obtaining specialty products. To specify the technical features of a biorefinery, design decisions are made along various development stages. At early-stages of development, the design space is broad and decisions involve high level *design variables*. As the biorefinery becomes more defined thorough pilot and demonstration testing, the design space gets narrower and the decision making involves more detailed variables. For example, for an early-stage design, a decision might concern the conversion process (e.g. fermentation as one alternative and thermochemical conversion as another), whereas in later stages of development the decision may be about conversion parameters for the chosen alternative (e.g. temperature, aeration rate). Decisions over a variable are made along iterative processes that include the generation and exploration of alternatives (e.g. calculations, simulations, experiments), and a final decision.

In biorefinery design practice, sustainability has mostly been approached from an engineering perspective. As part of assessment, integration, and optimization methods, sustainability is typically defined through metrics that indicate impacts on global warming and energy efficiency (Palmeros Parada et al. 2017). In this way, sustainability has been reduced to a few indicators that fit engineering methods but do little to address the complexity of the concept, as discussed above. To design for sustainability there is a need to open up to different methodologies and fields of knowledge (Azapagic and Perdan 2014) in order to address the contextual implications of biobased projects and the values of stakeholders on which different sustainability judgements are based (Asveld and Stemerding 2018). Value sensitive design (VSD), an approach to designing with consideration of stakeholder values in the context of a particular technology, is therefore a promising approach to the development of more sustainable biorefinery concepts.

### Value Sensitive Design

VSD is an approach to the design of technologies that proactively seeks to consider human values during the design process (Friedman et al. 2008a, b). With a focus on design projects, VSD is grounded on the understanding that the influence of a technology on society depends on its technical features, the context of its implementation, and its stakeholders (Davis and Nathan 2015). VSD is applied through three investigations: (a) a *conceptual investigation* to identify stakeholders and their values in relation to a technology, (b) an *empirical investigation* to recognize understandings and contexts concerning stakeholders’ values and the technology, and (c) a *technical investigation* that leads to the accommodation of investigated values in a design outcome (Friedman et al. 2008a, b). Iterations between these investigations, which are not necessarily independent, can serve to validate or gain more insight into how stakeholders’ values can be better supported by the technology.

Until now, VSD has mostly been applied for the design of artifacts and software, and its application for technological systems, such as biorefineries, is not easily deduced from previous experiences. This is largely because the development of biorefineries is a long process that requires the involvement of diverse stakeholders and large investments that cannot be reallocated once they have been made. This brings biorefineries into a complex socio-technical domain. Facing equally complex situations, VSD has been applied for the early stages of development of an urban simulation system (Borning et al. 2005; Friedman et al. 2008a, b), and to approach the energy transition in Finland (Mok and Hyysalo 2018). However, these cases greatly differ from that of biorefineries as they address already existing systems or specific parts of them (i.e. a city, a building), with clearly identifiable stakeholders and locations, and for which the design of the system itself was not in focus. Therefore, it is not possible to take a similar approach to the design of biorefineries. In biorefinery design there is no pre-existing system. Involving stakeholders can be problematic when they come from a variety of sectors and geographies, especially in the early stages of development when different products or feedstocks are still under evaluation. Even when revamping an existing industry, designing a biorefinery implies creating a system with new stakeholders related to, for example, new biobased products. This means that stakeholders’ roles and interests in a biorefinery can be very uncertain or tenuous, and involving them may require commitments that are not easily made at the early stages of a project.

The application of VSD for delimiting the design space of biorefineries in early stages of development has been explored recently (Palmeros Parada et al. 2018). As result, design propositions were derived as project specific design principles, and the authors suggest that reflecting about these propositions can support the integration of values in the subsequent design of biorefineries. In a similar line, Yoo et al. (2013) show that promoting reflection in a co-design space results in the identification of new technical features for the design of a value sensitive device. However, there is no experience on this type of work to derive a value sensitive technical concept, especially for a complex system like a biorefinery, and integrated to a design project. This leads to the observation that while various methods for value elicitation and empirical data analysis have been used in VSD (e.g. Czeskis et al. 2010; Dantec et al. 2009; Miller et al. 2007; Pommeranz et al. 2011), not much has been elaborated about the design process itself. There is some empirical work on the translation of values into design principles and desired technical features, and the works by Miller et al. (2007), and Xu et al. (2012) are particularly insightful. But no systematic investigation has been elaborated on the consideration of values during the technical design process, when alternatives are generated, explored, and decided over. This is not only a crucial point when defining technical features in support of stakeholders’ values, but such an analysis could also serve as a reference for future developments, as suggested by Oosterlaken (2015). Therefore, this work focuses on the generation and exploration of design alternatives, and how decisions about design variables are made in support of stakeholders’ values.

### Midstream Modulation

To bring reflection into biorefinery design practice, we looked at MM, a method that focuses on the practices of researchers and their decision making. MM is applied to broaden R&D practice to include considerations of societal aspects (Fisher and Schuurbiers 2013). MM is typically applied as a series of interventions that promote reflection and can result in the *modulation* of R&D decision making. When R&D participants improve their performance within the bounds of theories and values common in their field, it can be said that they are involved in a normal or de facto modulation of their practice (Fisher and Schuurbiers 2013). Then, as engagement takes place with an MM researcher, R&D participants are prompted to reflect upon their decisions and their potential impact, while becoming aware of themselves as agents in their own practice and of the de facto modulations. This *reflective*^1^ modulation has the potential to incite the envisioning of alternative paths in the participant’s practice (Fisher and Schuurbiers 2013; Schuurbiers 2011). Lastly, *deliberate* modulation has been recognized as a consequence of gained reflective awareness (Fisher and Schuurbiers 2013; Schuurbiers 2011), expressed as its deliberate use for the direction of decision making in R&D activities with consideration for societal aspects (Flipse et al. 2013).

Therefore, in contrast to VSD, MM does not explicitly aim to direct the outcomes of R&D activities towards a specific target, i.e. to integrate the values of stakeholders within the design concept, or support a central value such as safety. As Fisher and Schuurbiers (2013) put it, MM encourages reflection not to “shape the process” but rather to “stir” it. Nonetheless, MM has been shown to successfully raise levels of reflection and to result in a deliberate change of practices in R&D decision making, with considerations beyond those typical to R&D in both academic and industrial environments (Flipse et al. 2013; Schuurbiers 2011). Therefore, MM could also be applied to promote reflection about stakeholders’ values in a design context. Particularly, identified stakeholders’ values and design propositions, as derived for biorefinery design by Palmeros Parada et al. (2018), can be brought forward through MM interventions along the design process. Bringing forward these elements to a design group could promote reflection and support the identification of new technical features, as shown by Yoo et al. (2013) with stakeholder prompts. Then, by promoting reflection with MM during a design project, value considerations could be integrated into the biorefinery design process.

## Methodology

In this work, the consideration stakeholders’ values during the design of a biorefinery for bioplastics production was investigated. For this, MM was adapted to promote reflection about stakeholders’ values during the decision making processes over design variables. These values were identified following the work by Palmeros Parada et al. (2018), through a design space investigation. In the next paragraphs the case study and the followed methodology are presented in more detail.

### Case Study

#### Design Project

The development of a design project carried out from January to June 2017 was investigated. The project was developed by a design group participating in an international business competition for biobased production. This competition was organized by actors in the biobased sector, and was targeted towards graduate students with the aim of stimulating entrepreneurship and innovation in the biotechnology and bioengineering field. For the competition, the group had to develop a business plan for their own biorefinery concept; thus, they had to design not only a biorefinery, but also a plan of how to implement it as a business. The evaluation criteria for the competition were: design quality, business plan viability and originality, sustainability^2^ performance, and presentation of the business plan. However, no further detail was given with respect to these criteria. The prize of the competition was a grant to continue the research and development of their idea.

####  Design Group

The starting point of the design project was ongoing research at TU Delft on microbial platforms to produce a biodegradable biobased polymer (BBP), which is an alternative to fossil-based plastics. Because of the early stage of development of this technology, the design was at a conceptual level, where all available information was theoretical or experimental at lab scale only. The group was composed of two Process Design PDEng^3^ trainees (Designers 1 and 2, D1–D2) who had no previous connection to the research project, and a PhD candidate and two master students who worked on the larger research project in the university (Designers 3, 4 and 5, D3–D5). Most of the designers worked on the project as an additional activity to their regular work. Additionally, a group mentor with experience on biotechnology research supported the group during the project development. A sustainability team, composed of D1 and D4 was defined at the beginning of the project. The word team is used to refer to this subgroup in contrast to the whole group including all the designers.

#### Researcher Stance

The authors of this article are academic researchers focused on understanding societal and sustainability aspects of biotechnologies, and the use of this understanding in support of responsible innovation practices and communication processes. All authors work in the same research group and have experience with biorefinery design, life cycle and technology assessments, and midstream modulation. Although the authors work at the same university as the designers, they had not collaborated previously. The collaboration for this work started after the suggestion of the design group mentor, who was aware of the authors’ field of research. The first author, also referred to as the researcher throughout this paper, was in charge of the field work and had all contact with the design group. The first author is currently doing a doctoral dissertation and has research interests on how technological innovations in the fields of biotechnology and renewable energy can be developed responsibly and in support of sustainability.

### Activities

**Fig. 1:**
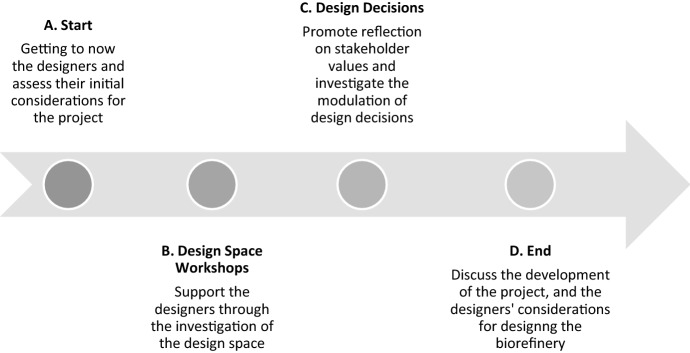
Schematic representation of the four different phases of this research

The development of the case study took place along four project phases schematized in Fig. 1. Throughout these phases, the researcher conducted 10 different activities with the design group as described in Table 1.

**Table 1:** Overview of activities along the project development

Phase	A. Start		B. Design space workshops^a^			C. Investigation of the design decisions				D. End
Time (weeks from first activity)	0	1	2	3	5	7	10	12	15	17
Activity^b^	Individual interviews	Group meeting observation	Workshop with sustainability team	Workshop with sustainability team	Workshop with sustainability team	(1) Interview with sustainability team	Interview with sustainability team	Interview with sustainability team	(1) Group meeting observation	Individual interviews
						(2) Group meeting observation			(2) Interview with sustainability team	
Designers present	D1–D5	D1–D5, mentor	D1, D4	D1, D4	D1, D4	(1) D1, D4	D1, D4	D1^c^	(1). D1, D2, D4, D5	D1–D4^d^
						(2) D1, D3–D5, mentor			(2) D1, D4	

^a^ After each of the activities in this phase, the designers investigated identified stakeholders (Table 3)

^b^Roman numerals indicate different activities in the same project time

^c^D4 was not available to participate

^d^D5 was not available to participate

####  Start and End Phases

Interviews at the start and end of the project were held with all group members, D1–D5. These interviews were prepared to identify changes in value considerations, as complement to the data gathered along the development of the project. These interviews also served to start identifying the main design variables in the project, and to investigate the expectations of the designers with this research. An interview guide was prepared (available in Supplementary Material) but the interviews were flexible, leaving room to explore emerging topics. Additionally, the information from the interviews was complemented with observation data from the first group meeting, where all group members discussed their ideas about the project.

#### Design Space Workshops

These workshops were intended to support the designers through the design space investigation as proposed by Palmeros Parada et al. (2018). The objective of this investigation was to identify potential stakeholders and their values, and to derive design propositions to guide later design activities. For this, three 2-hour sessions were scheduled with the sustainability team, and during these workshops the researcher asked questions and guided the sustainability team through a discussion about stakeholders, their values, and the relationship between the project and the identified values. Available notes and board photographs from these sessions, and documents exchanged with the team were used for subsequent analysis (see Supplementary Material).

First workshop: The sustainability team together with the researcher identified relevant stakeholders to the project. Using a generic biobased production chain as a starting point for discussion, the participants started to identify stakeholder groups that could be affected by the biorefinery and its development. All of these potential stakeholders to the future biorefinery are generically referred to as stakeholders, with no distinction between direct and indirect stakeholders as typically done in VSD, because they are all equally distant to this project or their role is uncertain in this early-stage of development. When necessary, however, ‘project stakeholders’ are specifically mentioned given that they are a clearly identifiable group of direct stakeholders at this stage of development (see “Design Space Workshops” in Results).

Second workshop: The initial findings about identified stakeholders were discussed in the context of the project, identifying where further research was needed. The discussion was focused on gathered information about the expectations, hopes and concerns of stakeholders (from, for example, the mission and vision of the identified organizations, their statements in relation to bioplastics and biobased production, and past actions or ongoing projects in the field). The information was based on public media and reports, academic literature, and, when possible, through direct contact with representatives from the identified organizations. As part of the competition, the team also had the opportunity to contact business professionals and academic researchers in the scope of the project or with experience in biobased production.

Third workshop: All the gathered information about the stakeholders was analyzed to identify values of relevance for the project. Once a value was identified, it was put into contrast with care for nature, intergenerational justice and distributive justice, as constitutive values of sustainability (Palmeros Parada et al. 2018). In this way, values from all stakeholders that related to sustainability in the context of this case were identified. Additionally, the sustainability team and the researcher conducted a preliminary examination of how the identified values were related to the technical aspects of the project. For this, the team made a block scheme of their project and marked the aspects that they considered to be related to a given value (e.g. a feedstock, a processing step). Based on this exercise, after the third workshop, the sustainability team developed a set of design propositions that suggested boundaries to the design space.

#### Investigation of the Design Decisions

MM serves as basis for the activities in the investigation the design decisions and as theoretical lenses for their analysis. For this, the researcher had four interviews with the sustainability team along the design project to promote reflection about stakeholders’ values. During these interviews the researcher asked the team to discuss the *alternatives* they were exploring for the *design variables*, the *considerations* involved in their decision making, and the *outcomes* they anticipated. This inquiry was based on previous MM literature, with two adaptations: (1) *research opportunities* investigated in MM were substituted by *design variables* to bring MM to the design context, considering that both imply potential paths for action that designers and researchers decide over; and (2) decisions were discussed in relation to the values and design propositions from the design space workshops, in contrast to the generic social and economic perspectives of previous studies (Fisher et al. 2006; Fisher 2007; Flipse et al. 2013; Schuurbiers and Fisher 2009). The researcher asked the designers about these aspects directly and indirectly, and prompted them to explain their assumptions, for example by pointing to extreme alternatives. Although the interviews were focused on the design variables being explored at the time, they remained flexible.

Additionally, the researcher took part in two group meetings attended by most of the group members and the mentor (see Table 1). These meetings were set up by the group, to share their results, discuss difficulties, and agree on subsequent activities. The researcher observed the discussions and, if time was available, asked questions about the group's design decisions, considerations, and expected outcomes.

### Data Analysis

The gathered data was coded and analyzed by the researcher to identify emerging values. Values identified from the start phase data were contrasted with the values from the design space workshop data, the decision making investigation, and from the group’s final report for the competition. This made it possible to analyze if and how the value considerations and reflections changed as the project progressed. For this, audio files, pictures, and notes were analyzed with the use of MAXQDA 12.

The identified values from the start phase (project values) and the design space workshops (workshop values) are presented separately in Results. The consideration of all of these values during the design of a biorefinery was investigated by analyzing the decisions about each project variable, and is presented in Investigation of the design decisions in Results. This analysis centered on the various decision making processes in which *alternatives* were being explored for defining the *design variables*. The identification of different modulation levels according to MM literature (i.e. de facto, reflective and deliberate, see “Midstream Modulation” in Background) allowed to recognize the emergence of reflection concerning specific issues of relevance to the project. Feedback on this analysis was received from several designers (D1, D3, D4) by email, or discussed in person.

## Results

### Start Phase

During the start phase, the design group defined their project by agreeing on the microbial platform and the main feedstock of the process. Sugarcane was decided upon as feedstock because of its high sucrose content (input for the microbial conversion). Brazil, as a leading country in the production of both sugarcane and biofuels, was selected as the target country for the project. Therefore, the definition of the feedstock type fixed the location of the design project: the sugarcane producing areas of Brazil. Once these aspects were agreed upon, four main design variables were discussed by the group: (1) feedstock streams, (2) products, (3) processing and technologies, and (4) business plan. These variables and the main alternatives considered during the decision making process are described in Table 2.

**Table 2:** Description of the four main design variables

Design variable	Description
Feedstock streams	This variable relates to the main input material for the biological conversion for obtaining BBP. From the beginning of the project the considered feedstock was sugarcane. Therefore, rather than addressing the feedstock crop, the group discussed whether to consider sugarcane juice only or whole sugarcane, as harvested. This choice relates to the first steps of sugarcane processing, which leads to two primary fractions: a sugarcane juice that contains most of the sucrose, and a bagasse fraction mostly composed of fibers
Products	Different product forms and by-products were considered as alternatives. Decisions over this variable ran parallel to some of the decisions about process and technology because certain products can only be obtained with certain technologies or process configurations. Also, the decision to consider the whole sugarcane as feedstock meant that sugarcane bagasse was available for processing. The main product alternatives were to produce pure BBP or a co-polymer of BBP with other compounds. Energy as a by-product from bagasse was also considered, as well as using bagasse for the production of compounds to co-polymerize with BBP (resulting in second generation or 2G copolymer compounds), and BBP itself (2G BBP)
Process	Different alternatives were explored in relation to the unitary operations of the production process, the process structure, and operation mode (i.e. continuous or in batches). The alternatives for the recovery of the product from the microorganism and its purification were widely discussed, i.e. whether it should be a mechanical, enzymatic or chemical process, or a combination. Also, when co-polymer products were being considered as alternatives, polymerization processes were included in the discussion
Business plan	The business plan was explored in relation to what the team considered to be their value proposition (e.g. lower production cost, biodegradability), their target clients (e.g. plastic producers, companies specialized in biodegradable plastic), and most significantly, the business model. Alternatives to the business model were related to the option of integrating the process and/or business with an existing sugarcane mill, running it as a partnership or licensing the design

Project values were identified at the start phase as the aspects that the group considered relevant for the project itself and for the competition (brief descriptions of all values are presented in Table 4). The majority are values typically associated with the science and engineering domains, such as process simplicity, scientific focus and technical feasibility. Achievement and designing feasibility appear to be more related to the competition context presented in the methodology section: The designers found it important to do what was needed to try to win the competition, taking their expertise and the available resources (e.g. time, data, and software) into consideration. Also, each of the designers mentioned sustainability as an important part of their project. Specifically, they spoke about the biodegradability of the target product as a means to prevent the pollution and harm to the environment that is typically associated with conventional plastics. Furthermore, they discussed that their project was about proposing an alternative to fossil resources, BBP being renewable and potentially associated with fewer CO_2_ emissions than conventional plastics. In other words, the designers discussed their project as a potentially more sustainable alternative.

### Design Space Workshops

In the first two workshops the team raised questions and discussed the implications of bioplastics production and use, the related stakeholders, and potential locations where production could take place. Starting from a generic production chain for biobased products, the sustainability team initially added two extra steps: the application production process (i.e. from bulk plastic to end-use products) and the waste management process. In this way, the designers made a distinction between the scope of the project for the production of a biobased plastic polymer, and its use as raw material for the production of end-use products such as packaging. Also, they observed that some stakeholders would vary according to the end-user’s location (e.g. would the plastic product be exported worldwide or would it be sold locally?). Another topic that was raised related to the food and sugarcane-ethanol industries. These industries were discussed as affected parties or even as potential participants in the production process. Once they had discussed the parties affected by the operation of the production chain, the discussion turned to stakeholders who could be affected or be involved during development, notably the public, NGOs, government and academia. For example, government bodies were discussed as the parties who set the rules and enter into commitments that could potentially open up a space, or could incentivize or discourage this type of technology. Table 3 summarizes the stakeholder groups that were identified and specifically investigated by the team.

**Table 3:** List of stakeholders investigated during the project

Stakeholder	Relation to biobased production or this project^a^
Biobased end-product manufacturers and industrial associations	They use bioplastic as feedstocks for the production of goods that reach final users (e.g. food-packages, eating utensils). (DC)
Bioplastic producers	They produce plastic materials from biomass resources, such as corn-based PET. (DC)
Biotechnology business experts	They manage capital investments and businesses in the field of biotechnology. (DC*)
Project stakeholders	This group includes the design group, researchers associated with the BBP research project, and the competition organizers. (DC)
Waste and recycling companies	They process waste streams to a desired quality, or to obtain new products. (DC*)
Bioethanol companies, logistics and industrial associations	They produce, distribute and/or purchase sugarcane, and produce bioethanol
Conventional plastic end-product manufacturers	They produce manufactured plastic goods from fossil resources, which reach a final user
Conventional plastic manufacturers	They use fossil resources for the production and/or transformation of plastics
Non-governmental organizations	They are related to the preservation and recovery of natural resources, and educational activities about bioplastics, their consumption, and environmental laws
Regional government	This group includes regional government branches in charge of developing plans and actions related to agricultural and industrial production

^a^DC stands for direct communication with representatives of the related stakeholder group, which includes face to face interviews and multi-media calls with the designers. An asterisk (*) indicates that the direct communication took place after the design space workshops

Several values were identified as a result of the design space workshops and the investigation of stakeholders by the design team, and are presented in Table 4. From this table it can be seen that, from the beginning, the design group as a whole was already familiar with many of the sustainability issues that were relevant to the project. Part of this awareness may be due to some of the designers’ academic experience with bioplastics (D3–D5), having had opportunities to face discussions about the sustainability of these materials. However, the design space workshops and related investigations were not redundant, as they served to understand these values more specifically for their case and considering the stakeholders (see Table 3).

**Table 4:** Values identified from the start phase and the design space workshops

Values	Start phase	Design space workshops^a^	Related stakeholders^b^
Achievement	The group’s ambition to win the competition	N/A	Project stakeholders
Biodegradation and environmental safety	Importance of cleanliness and prevention of harm to life (human and non-human), related to the persistence of waste, including plastic, the pollutants released, and chemicals used during production of biobased alternatives. Biodegradability as a desirable trait of the product. Since the material degrades over time, it is expected to be less harmful to the environment than “conventional plastics”	Broadened to considerations of the actual plastic biodegradation, with regards to the conditions under which the material degrades (dependent on its composition), and the extra processing and infrastructure required for it	Biobased end-product manufacturers, bioplastic producers, waste and recycling companies, NGOs, and project stakeholders
Cooperation	N/A	Cooperation in the sense of building partnerships with various organizations to ensure the success and long-lasting durability of the initiative	Bioplastic producers, bioethanol companies
Designing feasibility	Related to the group’s capacity to design a biorefinery and the business case with the available resources for the competition, especially time and data	N/A	Project stakeholders
Entrepreneurship	The need to design a cheaper process for bioplastics that can compete with conventional plastics in the market	The competition with conventional plastics as in Start Phase, and also about expanding the design and business idea to other applications and locations	Biotechnology business experts, biobased end-product manufacturers, bioplastic producers, bioethanol companies, conventional plastic producers, NGOs and project stakeholders
Food security	Using food crops for non-food applications is not desirable as it may affect food access for a section of the population. Awareness that that all crops, edible or not, use land and may affect food price or availability. Preference for second generation (2G) feedstock	No changes from the start phase	Biobased end-product manufacturers, NGOs and project stakeholders
Leadership	Developing a product and/or process that is inventive and that will contribute to companies’ market leadership	No changes from the start phase	Bioplastic producers and project stakeholders
Process simplicity	Minimum operational needs, such as lower maintenance requirements, or fewer conversion steps	N/A	Project stakeholders
Product quality	Generically related to a given quality or trait that is desirable in the product. At this point, it is vaguely related to ease of use, irrespective of the user	Further specified in the context of end-product applications, and considering the reliability of the design	Biotechnology business experts, biobased end-product manufacturers, conventional end-product manufacturers and project stakeholders
Renewability	The substitution of fossil-based resources with a renewable material, the importance of reducing CO_2_ emissions	No changes from the start phase	Biobased end-product manufacturers, bioplastic producers, bioethanol producers, NGOs, and project stakeholders
Resource efficiency	Minimum use of resources per unit of product. Focused on conversion yields, such as amount of substrate needed for the product	Broadened to using less of all resources (e.g. water, energy) in the production process	Biotechnology business experts, bioethanol companies, waste and recycling companies, NGOs and project stakeholders
Scientific focus	Related to the interest of the project stakeholders in investigating the “real-world” feasibility of the research being conducted in the university	N/A	Project stakeholders
Technical feasibility	Related to the design of an applicable process, one that works in practice	N/A	Project stakeholders

^a^N/A: the value was not discussed in the corresponding project phase

^b^Input from biobased business experts and waste and recycling companies is included although the team was only able to talk with them after the design space workshops

During the workshops the designers had the opportunity to think more deeply about many of the stakeholders they identified and investigated, as well as the emerging sustainability aspects in the context of the project. This was particularly evident on the subject of biodegradation. While the group had already expressed the importance of biodegradability during the pre-interviews, it became clear during the design space workshops that ensuring the actual degradation of the material was also important. This originated primarily from the investigation of non-governmental organizations who were critical about bioplastics, and who noted that while bioplastics were often advertised as more sustainable, little effort was done to ensure that they were biodegraded. This made the team recognize that some biodegradable plastics, depending on their composition, required specific conditions for their biodegradation, otherwise they could remain in the environment for a long period (see, for example, Emadian et al. 2017).

In the last session of the design space workshops the team reflected on the relationship between the identified values and the design variables. This occurred as the team derived design propositions to delimit the space for decision making. However, these propositions remained very generic. For example, in relation to environmental safety and resource efficiency the team proposed that the project should “ensure waste minimization by ensuring maximum utilization of raw materials and proper design selection”. This activity invited the team to think of the design project and their prospects for decision making in relation to all identified values and sustainability. While the contribution of the design propositions may initially appear negligible, in the next sections it will be presented how the results from the design space workshops served as modulators of the designing process.

### Investigation of the Design Decisions

The design group developed the project by making design decisions regarding four main variables (Table 2). The decisions for these variables were analyzed to see if and how the workshop values were being considered. Overall, six different decision making processes going through de facto*, reflective* and *deliberate* modulations were identified. To illustrate these decision making processes, an account of two of them is presented in this section, one about the main product of the project, and the second about the business model. In Table 5 these two processes are summarized with reference to the different modulations and considered values; all six decision making processes can be found in Supplementary Material**.**

**Table 5:** Identified modulations in the two decision making processes discussed in this section. Final decisions refer to what was discussed as their design concept by the end of the project

Modulation	Alternatives and decisions^a^	Considered values
*Decision making process I—products*		
De facto	Bagasse as feedstock for energy production (1)	Achievement, process simplicity, resource efficiency, technical feasibility
	Pure BBP (1)	Scientific focus
Reflective	Bagasse as 2G feedstock (5)	Achievement, entrepreneurship, food security, resource efficiency
	Co-polymers as main product (5)	Achievement, entrepreneurship, product quality, resource efficiency
Deliberate	Investigate alternative uses for bagasse: 2G BBP production (5)	Achievement, entrepreneurship, food security, product quality, resource efficiency
	Investigate co-polymer compound alternatives and their production (5)	
Final decision	Model energy generation from bagasse and the production of pure BBP granules. The sustainability team suggested to include a proposal in the business plan to research 2G and wastewater BBP production	All of the above
*Decision making process II—business model*		
De facto	The model has to be able to accommodate the integration of the process with an existing sugarcane mill: a partnership with an existing sugarcane mill was the group’s initial idea to achieve this (7)	Achievement, cooperation, entrepreneurship, resource efficiency
Reflective	The integrated process can be supported by other business models: BBP production as part of the same sugarcane company (merge model), or by licensing the patented technology to the mill companies (licensing model) (7)	Cooperation, entrepreneurship, resource efficiency, growth, leadership
	If the business is integrated, there is an option to vary the production of any of the products as desired (10)	Entrepreneurship
	A licensing model implies confidentiality until a patent application is made (12)	Scientific openness
	A licensing model can be combined with a biodegradation step (12)	Biodegradation
	The merge model means losing ownership of the project (15)	Ownership
Deliberate	Discard the licensing alternative and investigate further into the stand-alone and partnership business models (15)	Entrepreneurship, scientific openness
Final decision	Partnership model for the business	All of the above

^a^Project time of first observation in parenthesis; see Table 1 for more details of the project phases

#### Products

Pure BBP, the main biorefinery product, was the starting point for the products variable. Additionally, after deciding to process the whole sugarcane, bagasse (i.e. a sugarcane processing residue) was recognized by the group as an available material that could be used in the biorefinery to produce electricity. So, energy became their de facto alternative as a by-product. However, during the 3rd workshop with the sustainability team (week 5), the assumption that the product would be pure BBP granules and that bagasse would only serve for energy generation was questioned. The sustainability team *reflected* over different possibilities they identified for the main product. Particularly, they spoke about a co-polymer as a main product (i.e. the product could be composed of two types of compounds, the original BBP and a second co-polymer compound). The group argued that a co-polymer product would have better properties and consequently a higher market price. When speaking about one of the possible compounds for the co-polymerization, the team recognized that they had the option to use bagasse to produce it. Using bagasse for producing a co-polymer compound was expected to appease concerns over food security and first-generation production (i.e. a part of the plastic product would be produced from a non-food raw material). This discussion resulted in the exploration of using bagasse for producing a co-polymer compound and also for producing second generation^4^ (2G) BBP from a different metabolic route.

However, during the first interview with the sustainability team (week 7), after enquiring again about the utilization of bagasse, the sustainability team explained that they had decided, after exploring other alternatives, to design for energy generation*.* In this case, the team *deliberately* changed their original idea: they explored and researched alternatives based on considerations that reflected the workshop values. However, due to their concerns over their own expertise and the feasibility of designing for the 2G alternatives, as well as the impact it would have for the competition, they decided to keep to their de facto idea (i.e. production of BBP granules and bagasse for energy). Nevertheless, the sustainability team stated that the explored alternatives would be integrated in the final report as an alternative to consider for future research, writing in the groups’ final report: “Another interesting solution would be the simultaneous studies of using the sugarcane bagasse for the production of PHB… This will ensure we are not dependent on sugarcane juice alone and will also reduce the competition with sugarcane used as a food source, which is part of our sustainability design proposition.”

#### Business Model

The business model discussion was started later than the other variables. It was first observed during the first interview with the sustainability team (week 7) when the group was already investigating the production process as integrated with an existing sugarcane mill. Their de facto idea about the business model was then related to this decision only: the business model had to be able to accommodate the integration of the process. The initial alternative of the group was to have two separate companies with integrated streams. This integration would take place by means of a type of partnership that would allow to buy and sell each other raw materials and utilities. However, later in week 7 they *reflected* about other possibilities for developing the integration idea: The alternatives to the partnership model were to merge their business into a milling company, and a licensing model in which they would not sell the BBP product, but rather license the technology.

The group discussed these options while reflecting on their implications in the interviews in weeks 7, 10 and 12. They concluded that the partnership model would benefit their project by increasing resource efficiency, while providing engagement for cooperation and business growth. Regarding the merging model, they also anticipated a positive effect on resource efficiency, and flexibility for the product portfolio. As for the third alternative, they thought that the licensing model would allow them to cooperate with multiple companies and to generate opportunities for expanding the business idea. Additionally, they discussed the idea that licensing agreements might offer the option of pushing for a biodegradation deal with licensee companies.

However, by week 15, the team *deliberately* focused on the partnership model, and discarded the license idea due to its undesirable implications for their project. Aiming for a licensing model in the long term seemed to the team to be too risky when considering the high competition in the bioplastics market. More crucial to the discussion, however, seemed to be the realization that a licensing path conflicted with the scientific openness endorsed by the project stakeholders. Also, the sustainability team discussed how the merging model would imply a loss of ownership of the production process and the project. In this way, ownership of the project and scientific openness were discovered to be project values that had not been recognized before, either by the group or the researcher.

Having an increased awareness of the values at play, the group deliberated over the alternatives and the values that they did or did not support. As a final decision for the business case, the group decided to favor the alternative that better suited the scientific pursuits related to the project and the feasibility of starting it up as a business. However, having disregarded the option that could support biodegradation, the team explained they would support the value in another part of the project, i.e. through the targeting of clients (see Supplementary Materials for more details on this decision process). Specifically, D1 stated during the interview in week 15*: *“We need to focus on companies that are looking for this kind of sustainable solutions or biodegradable plastic, and then target them as customers to make sure that it actually goes where it has to end up”, referring to the plastic biodegradation.

## Discussion

In this section the integration of stakeholders’ values as part of the design project is discussed. Firstly, we discuss if identified stakeholders’ values were considered during the design process. We also discuss how bringing forth the investigated values and design proposition during the design decisions supported this consideration. Subsequently, we discuss this exploratory work in the context of VSD. We suggest that the present work can be applied as a technical investigation in VSD. We then argue that bringing elements of VSD into biorefinery design practice with flexibility, considering what is possible in the project, can serve to bring value considerations during their development. Next, the role of the researcher in this work is discussed in contrast to typical MM and VSD literature. Lastly, some implications and limitations of the presented work and recommendations for future studies are presented.

### Reflection and Value Integration in the Design Process

Value considerations changed along the development of the project. With the start phase activities it was possible to see that the group was generally aware of most of the values found to be relevant to the project. The understanding of these values, however, became richer during the design space workshops when the team gained awareness of how different stakeholders cared about different aspects of the project, most prominently in the case of biodegradation. Already at this point the team started to reflect somewhat about the relationship between the values and their design project, however this understanding was still vaguely specified for their project as shown with the design propositions (see “Design Space Workshops” in Results). It was during the investigation of the design decisions, as the team advanced in their project, that this relationship was put into focus.

The investigation of the design decisions was planned with the aim to encourage reflection and support the integration of values in the design decisions. It was found that the interviews in this project phase stimulated the team to reflect upon the ongoing design decisions and their relation to identified stakeholders and values. Although the design propositions themselves were not directly followed or considered to the letter for raising reflection (i.e. they were mostly too broad or not applicable), asking the team about them, and the identified stakeholders and values, stimulated them to envision new design alternatives. For example, while discussing the decision to implement an integrated process with an existing sugarcane mill, the group identified different alternatives for their business model that could support this integration, as well as how these alternatives related to the investigated values (e.g. licensing the technology and merging with a sugarcane mill). Furthermore, it was found that encouraging reflection during design decision making meant that the team remained open to the discovery of previously overlooked values that were relevant for the project. Scientific openness and ownership of the project are two examples, as elaborated in Results.

In occasions, value tensions emerged when the designers had to make decisions over design alternatives. As the team was prompted to talk about their decisions, they reflected on how the alternatives to a variable supported or opposed values in the context of the project. As result, the team became aware of emerging tensions, when choosing one alternative for a variable supported a given value but could undermine or negate the support to another value. Then, with a close understanding of the design space, the team had the opportunity to generate new alternatives and find solutions according to the specific decision at hand. For example, for the product recovery process, the values of efficiency, environmental safety, profitability, and quality were in tension. When looking at the emerging tension and the alternatives at hand, the team saw it was possible to combine two seemingly opposing alternatives: with the use of mild solvents in minimum quantities and in combination with a secondary processing, they would maintain a relatively low environmental risk and prevent large losses in efficiency, quality, and profitability. In this way, it seems the group intuitively followed the maximin principle (i.e. a decision rule based on the selection of the alternative that is best when looking at the least supported values of all alternatives van de Poel 2014).

However, it was not always possible to find new alternatives that eased value tensions. Particularly, when there was tension between project and workshop values, and there was no effective alternative, the final decision would tend to favor project values (especially regarding technical feasibility). When this was the case, it was observed that the team nevertheless sought to integrate the workshop values in other parts of the project. For example, once it became impossible to support biodegradation with their decision on the business model, the team proposed to target specific clients for their business case as shown in Results. They decided to focus on industries that would not only be interested in using biobased plastics, but that could also have an interest in their biodegradation (such as single-use plastic users who could accommodate industrial biodegradation within their business). Another example is related to using bagasse for 2G production. Although 2G production was perceived as having less risk for food security than 1G, the group found its feasibility questionable under the project circumstances. As a result, the group chose to focus on 1G production for their design, while 2G feedstocks were suggested for consideration in later research. These examples show that although project values were favored by the team for specific decisions, they still tried to accommodate the workshop values within the project. These values also seemed to have become a part of the project even in a context that was not specifically supportive, as indicated in the comment by D1 (week 10): “In the competition they said ‘why do you care? You’re producing the [bioplastic] granules and if people are ready to pay, then you give it and you don’t worry about where it ends up.’ But […] we want to make sure that […] [the granules] end in the right places and the plastic actually degrades”.

Through the design decision activities value considerations were then brought to the design desk. In this way the team was encouraged to find creative ways to integrate values in the design, not only for specific variables, but in the context of the whole project. This real-time response in the design process was possible as the designers had the capacity to find new alternatives and flexibility to deal with value tensions in accordance with each decision, instead of choosing values a priori or relying on a single solution strategy (e.g. cost–benefit or multi-criteria analyses) that can result in undesirable or unfeasible solutions (van de Poel 2014). Even more, it could be argued that facing value tensions without a predefined decision making strategy opened up a path for innovation, as suggested elsewhere in the literature (van den Hoven et al. 2012). Overall, a reflective design decision making, with openness to discover new values and technical features, and a flexible approach to value tensions is suggested as a good practice for integrating values in the design decision making processes.

### Contribution to Value Sensitive Design

The present work indicates that an investigation of the design decisions with MM could be applied as a technical investigation in VSD, which typically focuses on how technical features support or hinder human values, or the proactive value sensitive design of a technology (Friedman et al. 2008a, b; Friedman et al. 2017). In this work, the investigation of the decision making allowed to explore with the designers how design alternatives supported or hindered the identified values, and ultimately supported the integration of these values in the design decisions, as discussed above. Additionally, the analysis of the design decisions provided a record of the alternatives that were considered, and the reasons on why they were or were not taken for the project (see Supplementary Material). As discussed by Oosterlaken (2015), such a record can be part of a ´design library´ that inspires or informs the development of other biorefineries or technologies, and thus facilitates the integration of values in design. This record could also serve as self-reference for the researchers and developers of the same technologies, to look back to their decisions when evaluating and improving the technology in more advanced stages.

Furthermore, the present exploration on value considerations in design shows an example of how elements of VSD (e.g. the identification of stakeholders and values, and their translation to technical features) can be put to practice for the early stages of development of complex systems like biorefineries. Particularly, as previous VSD experiences in the literature were found unsuitable for the current biorefinery project, in this work some elements of the VSD investigations were integrated into common biorefinery design practice. This integration was done from the definition of the design space, with the design space workshops aiming for the identification of stakeholders and relevant values to the project, to the investigation of the decision making, with the analysis of how values relate to project variables and their consideration for producing a design concept (see Fig. 1).

It is acknowledged, however, that there are limitations on how these VSD elements were brought into this biorefinery design project. Particularly, while a design space investigation could serve to address aspects of the conceptual and empirical investigations of VSD (Palmeros Parada et al. 2018), in this exploratory work it was only possible to do so to a very limited degree. That is, there was no dedicated value elicitation method, and engagement with all identified stakeholders was not possible. This is a large contrast to VSD literature, where close engagements with stakeholders are a main aspect of applying VSD, allowing to, for instance, elicit values, identify desirable technical features, and address value tensions (e.g. Miller et al. 2007; Yoo et al. 2013). In this design project, for instance, the variables to the project were too broad to ask specific questions as in a survey, and the amount of reachable stakeholders would have been too limited to take their responses as a rule for choosing between alternatives. Additionally, in this work, there was not a clear difference between direct stakeholders and indirect stakeholders to the biorefinery. This is because, besides the project stakeholder, all other stakeholders were distant and uncertain in their potential role with a biorefinery at such early-stage of development (see Methodology). Therefore, the investigations along the design space workshops provided only an indication of the stakeholders and values relevant to the biorefinery.

Nevertheless, it is significant that at such an early-stage of development, reflection was started on identified stakeholders, their values, and the broader socio-technical context of the project. As a consequence, bioethanol organizations, for instance, were identified as potential stakeholders with whom to enter into a cooperative relationship. Also, the investigation into non-governmental organizations led the team to question their initial assumption about the positive impacts of biodegradable plastics. They realized that it was not only about designing for biodegradability, but also about ensuring the effective biodegradation of the material. Furthermore, they recognized themselves and other actors (e.g. end-product manufacturers, users, waste and recycling companies and the government) as parties that had a role in encouraging such biodegradation.

Therefore, bringing elements of VSD into biorefinery design practice with flexibility, considering what is possible in the project, can serve to bring value considerations to the design of complex systems like biorefineries along their development. Although bringing VSD elements to early-stage biorefinery design may face some limitations as presented in this case, opening a discussion about stakeholders and values can already contribute to the development of complex systems that are more responsive to emerging societal concerns. This achievement would be significant for biorefineries specifically, considering the ongoing debates around biobased production, as discussed in the introduction. Also, while in the presented case there was limited engagement with stakeholders, the integration of VSD elements to biorefinery design practice, as shown here, leaves room for conducting dedicated empirical investigations with stakeholders (as in the work by Palmeros Parada et al. (2018), or in-between design space workshops). This could be applied in more advanced projects with defined features (e.g. with a specific location or product application) that allow to recognize and engage stakeholders. In this way, VSD could be brought to the design of biorefineries with flexibility, depending on the design context as recently suggested elsewhere (Friedman et al. 2017), and considering the development stage of the project.

### The Role of the Researcher and the Designers

The role of the researcher is an important point of discussion that emerged during the development of this work. This is related to the two different approaches that served as basis to the present work, and in which the researcher takes different roles. MM researchers are in the field, they have frequent contact with participants but typically act from an independent position to the ongoing research (e.g. as embedded humanist in a research laboratory, Fisher et al. 2015). The role of the VSD researcher ranges from that of a researcher investigating the specific value implications of a technology for its design (e.g. Czeskis et al. 2010; van Wynsberghe 2013), to that of a designer or participant within a design group that investigates and takes these implications into account to design it (e.g. Miller et al. 2007; Xu et al. 2012). Therefore, in MM, the researcher has no aim to change a technology or research in a particular direction, nor the capacity to do so directly; in VSD the researcher has the aim to change the technology in consideration of stakeholders’ values, and can influence it directly (as a designer or part of a design group) or indirectly (e.g. by suggesting principles, guidelines, etc.).

In this work, MM was applied to promote reflection on stakeholders and the investigated values, to seek its integration in a design concept. Therefore, from the conception of this work, the role of the researcher was more similar to VSD, seeking to integrate stakeholders’ values in the design decisions for a biorefinery concept. However, the researcher remained somewhat external to the group, as in MM, not participating in calculations or making design decisions, for example. Additionally, although the researcher supported the team in the identification of stakeholders and on the analysis of how values related to the technology, the contact with stakeholders and the gathering of information was performed exclusively by the designers. Therefore, although the first part of this project (i.e. investigation of the design space) is closer to VSD in content and aim, the researcher acted from a more distant position to the design project than commonly for a VSD researcher.

As result, the designers had a more active role in the discovery of values and their translation into technical features than the researcher. This is a result of how the design project was set-up, with the designers as the registered participants of the competition, and the researcher with limited availability for participation. It was observed that this active involvement by the designers contributed to the reflective process, even in the early parts of the project, as mentioned in Results. However, in this case, not having the time or space for action resulted in limited capacity on the part of the researcher to investigate in depth the relationships between values and design decisions in areas where the designers had no expertise. This is particularly the case with complex issues such as food security, which remained a difficult aspect to deal with within the project. Therefore, having a dedicated VSD researcher with capacity to investigate value considerations and their translation into design features, as in the VSD cases of Miller et al. (2007), and Xu et al. (2012), together with a design group that is actively involved in the conceptual and empirical investigations of VSD and is encouraged to reflect upon the ongoing design decisions, as shown here with MM, is suggested for future work.

### Other Implications, Limitations, and Recommendations

By promoting a reflective design practice, the present work not only had an impact on the resulting biorefinery concept, as typically aimed with VSD (Doorn et al. 2013), but also brought a potential influence on the research trajectory related to the technology in question (microbial platforms). The discussions about feedstocks and products are an illustration of this, when alternatives that better supported the workshop values but that were considered unfeasible for this present project were still reported as aspects to consider in future developments. Although the extent of this influence is not known and cannot be proven in this work, it shows that reflective design exercises as presented here could be applied to open on-going research to societal concerns when researchers seek to explore the potential applicability of their work. Even more, applying such a reflective design approach in industrial environments could contribute to overcome the challenge of aligning responsible innovation and industrial practices (Dreyer et al. 2017).

However, having integrated stakeholders’ values in the development and design of a biorefinery does not mean that the outcome will be acceptable in societal terms. This limitation is related to the scope and forward-looking nature of the present work, and VSD too when applied in the context of biorefineries with long development times. Firstly, a technology can always be used differently than intended or anticipated by designers (Ihde 2008), and the farther in time a design is from the ultimate application, the more limited the capacity for anticipation will be. Secondly, even if all stakeholders had their values reflected in a design, there are other factors beyond the scope of a biorefinery design project that can shape its development, and thus its relation to stakeholders (e.g. governmental programs and policies, Bosman and Rotmans 2016). To illustrate these two points, we refer to the early phases of the biodiesel case in Brazil in the early 2000s: Biodiesel biorefineries and a diesel program were set-up to promote social development, but their initial result was a large participation from large-scale soybean oil producers and little inclusion of small-scale family farmers (Castellanelli 2016). Biorefinery operators mostly bought soybean oil (unanticipated), while the biodiesel program was not sufficient to incentivize the entry of family farmers to the fuel market (institutional scope beyond the biorefinery). In addition to the two previous points, the social, moral, and institutional context surrounding a technology can change with time and render a design with value considerations inadequate, as discussed by Asle H. Kiran in the scope of Responsible Innovation (2012). A broad example is the case of biofuels, which were initially regarded as sustainable because they are produced from renewable sources. Nowadays it is not enough that biofuels are renewable; other aspects like biodiversity and food security are recognized as important too as mentioned in Introduction.

Continuous learning about stakeholders and the context of a system or technology, like biorefineries, throughout its development and implementation can be an appropriate measure as suggested by Asveld and Stemerding (2018). In this way, the work presented here, seeking to contribute to the integration of stakeholders’ values during biorefinery design, can be applied as part of a continuous learning process about the societal implications of a technological innovation. Such a process could be applied, for example, as a broader iterative VSD practice, from early-stage conceptual design to more detailed stages. Also, in later stages when applications and stakeholders are more certain and easily involved, stakeholders could be involved by means of participatory evaluations (Borning and Muller 2012), or the selection of indicators (Dale et al. 2015), for example.

Finally, the applicability of this type of work in the industry may be put into question given the time, expertise, and commitment required to include it in the development of design projects. In this case, it helped to be embedded in a university environment, considering that some of the designers were involved in the research about the microbial platform and had an interest in learning more about their technology. An option to facilitate its industrial application could be the development of a framework that aligns VSD elements, not only with an overarching design approach, but also with common biorefinery design methods. However, such a framework would need to compatible with the industrial sector where specific conditions may pose a challenge to its application (e.g. confidentiality). Another avenue is to investigate whether recurrent VSD exercises can lead to knowledge about stakeholders and their values related to specific technologies and application contexts. This knowledge could serve to create a “design library” as mentioned before, and potentially simplify its application.

## Conclusions

In this paper we present an exploration of how values can be integrated into the decisions of a design project to obtain a biorefinery concept. This integration was approached by promoting reflection during the design decision making with MM. Particularly, MM interventions allowed to bring reflection over the variables of the project, and how design alternatives related to the identified values. This reflection allowed to generate new design alternatives, and to recognize and respond to emerging value tensions. In this way, we suggest that the present work can serve as basis for a systematic technical investigation of VSD, especially in the context of biorefineries.

Additionally, based on this exploration we conclude that, not only reflection, but also flexibility and openness are important for the value sensitive design of biorefineries. MM was proven useful to put this into practice, showing that an open and reflective decision making, with the capacity to adapt the design, gave opportunities to integrate values in design decisions and face emerging value tensions. For a value sensitive design of biorefineries and other complex systems we suggest to apply VSD with flexibility, in alignment to design practices in the field and considering the development stage of the project. Also, by looking at the role of the researcher in this work, we suggest that VSD should be applied by dedicated VSD researchers as part of a design group, where all designers are actively involved in the conceptual and empirical investigations of VSD and are encouraged to reflect upon their ongoing design decisions as shown with MM.

The presented work allowed to recognize and discuss emerging value tensions and contextual implications that are not usually part of the design process of biorefineries. While it is acknowledged that not all moral and societal issues can be solved, we suggest that this type of activity can be intended as part of a continuous learning process during the development of technologies and technological systems like biorefineries. However, there are some issues to be resolved regarding the applicability of such an approach in an industrial context, where confidentiality, for instance, could be detrimental to its objectives. Overall, by opening the design process to considerations of stakeholder values and societal concerns, the authors hope to contribute to the development of more sustainable biorefineries.

## Electronic supplementary material

Below is the link to the electronic supplementary material.

Supplementary file1 (DOCX 298 kb)

## Footnotes

1 In this work the word *reflection* is used in reference to both *reflection* and/or *reflexivity* to avoid unnecessary complexity.

2 Sustainability was stated as an important criterion in the competition, however no specific list of aspects to consider was given beyond stating its economic, environmental and social dimensions.

3 PDEng: Professional doctorate in engineering is a title given to graduate engineers who work on design projects for 2 years, typically in industry and academia partnerships. (“PDEng programmes” n.d.).

4 *Generations* are used to refer to the type of feedstock used for production: first generation refers to sugar or oil-rich process crops, while second generation refers to more recalcitrant lignocellulosic materials. Very often, first-generation crops are food crops and second-generation crops are considered residues or ‘energy crops’.

## References

[CR1] Asveld L, Stemerding D, van de Poel I, Lotte A, Mehos DC (2018). Social learning in the bioeconomy, the ecover case. New perspectives on technology in society: Experimentation beyond the laboratory.

[CR2] Azapagic A, Perdan S (2014). Sustainable chemical engineering: Dealing with “wicked” sustainability problems. AIChE Journal.

[CR3] Bauer F, Coenen L, Hansen T, McCormick K, Palgan YV (2017). Technological innovation systems for biorefineries: A review of the literature. Biofuels, Bioproducts and Biorefining.

[CR4] Borning A, Friedman B, Davis J, Lin P, Gellersen H, Schmidt K, Beaudouin-Lafon M, Mackay W (2005). Informing public deliberation: Value sensitive design of indicators for a large-scale urban simulation. ECSCW 2005.

[CR5] Borning, A., & Muller, M. (2012). Next steps for value sensitive design. In *Proceedings of the SIGCHI conference on human factors in computing systems* (pp. 1125–1134)*.* New York: ACM. 10.1145/2207676.2208560.

[CR6] Bosman R, Rotmans J (2016). Transition governance towards a bioeconomy: A comparison of Finland and The Netherlands. Sustainability.

[CR7] Castellanelli CA (2016). Los mecanismos de inclusion social: aspectos controversiales en el programa nacional de produccion de biodiesel en Brasil. Holos.

[CR8] Czeskis, A., Dermendjieva, I., Yapit, H., Borning, A., Friedman, B., Gill, B., et al. (2010). Parenting from the pocket: Value tensions and technical directions for secure and private parent-teen mobile safety. In *Proceedings of the sixth symposium on usable privacy and security* (pp. 15:1–15:15). New York: ACM. 10.1145/1837110.1837130

[CR9] Dale VH, Efroymson RA, Kline KL, Davitt MS (2015). A framework for selecting indicators of bioenergy sustainability. Biofuels, Bioproducts and Biorefining.

[CR10] Dantec, C. A. L., Poole, E. S., & Wyche, S. P. (2009). Values as lived experience: Evolving value sensitive design in support of value discovery. In *Proceedings of the SIGCHI conference on human factors in computing systems* (pp. 1141–1150). New York: ACM. 10.1145/1518701.1518875

[CR11] Davis J, Nathan LP, van den Hoven J, Vermaas EP, van de Poel I (2015). Value sensitive design: Applications, Adaptations, and critiques. Handbook of ethics, values, and technological design: Sources, theory, values and application domains.

[CR12] Doorn, N., Schuurbiers, D., van de Poel, I., & Gorman, M. E. (2013). Early Engagement and new technologies: Towards comprehensive technology engagement? In N. Doorn, D. Schuurbiers, I. van de Poel, & M. E. Gorman (Eds.), *Early engagement and new technologies: Opening up the laboratory* (Vol. 16, pp. 233–251). Dordrecht: Springer Netherlands. 10.1007/978-94-007-7844-3_12.

[CR13] Dreyer M, Chefneux L, Goldberg A, von Heimburg J, Patrignani N, Schofield M, Shilling C (2017). Responsible innovation: A complementary view from industry with proposals for bridging different perspectives. Sustainability.

[CR14] Emadian SM, Onay TT, Demirel B (2017). Biodegradation of bioplastics in natural environments. Waste Management.

[CR15] Fisher E (2007). Ethnographic invention: Probing the capacity of laboratory decisions. NanoEthics.

[CR16] Fisher E, Mahajan RL, Mitcham C (2006). Midstream modulation of technology: Governance from within. Bulletin of Science, Technology & Society.

[CR17] Fisher E, O’Rourke M, Evans R, Kennedy EB, Gorman ME, Seager TP (2015). Mapping the integrative field: Taking stock of socio-technical collaborations. Journal of Responsible Innovation.

[CR18] Fisher E, Schuurbiers D, Doorn N, Schuurbiers D, Van de Poel I, Gorman ME (2013). Socio-technical integration research: Collaborative inquiry at the midstream of research and development. Early engagement and new technologies: Opening up the laboratory.

[CR19] Flipse SM, van der Sanden MCA, Osseweijer P (2013). Midstream modulation in biotechnology industry: Redefining what is “Part of the Job” of researchers in industry. Science and Engineering Ethics.

[CR20] Friedman, B., Borning, A., Davis, J. L., Gill, B. T., Kahn, P. H., Kriplean, T., et al. (2008). Laying the foundations for public participation and value advocacy: Interaction design for a large scale urban simulation. In *Proceedings of the 9th annual international conference on digital government research*. Montreal: DG.O.

[CR21] Friedman B, Hendry DG, Borning A (2017). A survey of value sensitive design methods. Foundations and Trends® in Human-Computer Interaction.

[CR22] Friedman B, Kahn PH, Borning A, Himma KE, Tavani HT (2008). Value sensitive design and information systems. The handbook of information and computer ethics.

[CR23] Ihde D, Vermaas PE, Kroes P, Light A, Moore SA (2008). The designer fallacy and technological imagination. Philosophy and design, from engineering to architecture.

[CR24] Kiran AH (2012). Does responsible innovation presuppose design instrumentalism? Examining the case of telecare at home in the Netherlands. Technology in Society.

[CR25] Miller, J. K., Friedman, B., Jancke, G., & Gill, B. (2007). Value tensions in design: The value sensitive design, development, and appropriation of a corporation’s groupware system. In *Proceedings of the 2007 international ACM conference on supporting group work* (pp. 281–290). New York: ACM. 10.1145/1316624.1316668.

[CR26] Mok L, Hyysalo S (2018). Designing for energy transition through value sensitive design. Design Studies.

[CR27] Oosterlaken I (2015). Applying value sensitive design (VSD) to wind turbines and wind parks: An exploration. Science and Engineering Ethics.

[CR28] Palmeros Parada M, Asveld L, Osseweijer P, Posada JA (2018). Setting the design space of biorefineries through sustainability values, a practical approach. Biofuels, Bioproducts and Biorefining.

[CR29] Palmeros Parada M, Osseweijer P, Posada Duque JA (2017). Sustainable biorefineries, an analysis of practices for incorporating sustainability in biorefinery design. Industrial Crops and Products.

[CR30] PDEng programmes. (n.d.). Retrieved 18 January, 2019 from TU Delft website. https://www.tudelft.nl/en/education/programmes/post-academic-professionals/pdeng-programmes/.

[CR31] Pfau S, Hagens J, Dankbaar B, Smits A (2014). Visions of sustainability in bioeconomy research. Sustainability.

[CR32] Pommeranz A, Detweiler C, Wiggers P, Jonker C (2011). Elicitation of situated values: Need for tools to help stakeholders and designers to reflect and communicate. Ethics and Information Technology.

[CR33] Rosegrant MW, Msangi S (2014). Consensus and contention in the food-versus-fuel debate. Annual Review of Environment and Resources.

[CR35] Schuurbiers D (2011). What happens in the lab: Applying midstream modulation to enhance critical reflection in the laboratory. Science and Engineering Ethics.

[CR34] Schuurbiers D, Fisher E (2009). Lab-scale intervention. Science & society series on convergence research. EMBO Report.

[CR36] van de Poel, I. (2014). Conflicting values in design for values. In J. van den Hoven, P. E. Vermaas & I. van de Poel (Eds.), *Handbook of ethics, values, and technological design* (pp. 1–23). Springer, Dordrecht. 10.1007/978-94-007-6994-6_5-1.

[CR37] van den Hoven J, Lokhorst G-J, van de Poel I (2012). Engineering and the problem of moral overload. Science and Engineering Ethics.

[CR38] van Wynsberghe A (2013). Designing robots for care: Care centered value-sensitive design. Science and Engineering Ethics.

[CR39] Xu H, Crossler RE, Bélanger F (2012). A value sensitive design investigation of privacy enhancing tools in web browsers. Decision Support Systems.

[CR40] Yoo, D., Huldtgren, A., Woelfer, J. P., Hendry, D. G., & Friedman, B. (2013). A value sensitive action-reflection model: Evolving a co-design space with stakeholder and designer prompts. In *Proceedings of the SIGCHI conference on human factors in computing systems *(pp. 419–428). New York: ACM.

