# Supplementary material for: Integrating Value Considerations in the Decision Making for the Design of Biorefineries
Source: Sci Eng Ethics. 2020 Jul 7;26(6):2927–55. doi: 10.1007/s11948-020-00251-z (PMC7755630; doi:10.1007/s11948-020-00251-z)
Supplement: Supplementary file 1 — Supplementary file1 (DOCX 298 kb) [file 11948_2020_251_MOESM1_ESM.docx]

Supplementary Material

[I. Pre- and post-interview guide i](#_Toc17210307)

[II. Design Space Workshops iii](#_Toc17210308)

[III. Identified decision making processes iv](#_Toc17210309)

## Pre- and post-interview guide

This interview guide was used for the interviews at the beginning and at the end of the design project (see Table 1, discussed in section 3.2 in the main text).

**About the project**

1. Please tell me more about your project. What are the project goals?
2. Could you tell me about the origin of this thesis/competition project, where does it fit with the institution that organizes it?
3. Is this project part of another project? How is the larger project/competition funded? Is there cooperation with other universities, industry?
4. Are there any specific sustainability goals linked to your project?

**Scientific/technical significance**

1. What are currently the most important scientific/technical challenges of your project?
2. How does the project contribute to progress in your field of science and engineering?

**Project Progress**

1. How is the research reviewed during the research process? Who is involved?
2. Regarding decisions on research progress: who decides the direction of research? Subsequent steps? What is your role in this process?
3. What kinds of considerations play a role in these progress decisions?
4. What are the criteria for determining the success of your specific project?

**Expertise and roles**

1. What is your background? What is the background of others in the development of this project?
2. Do you think there should be more people with different expertise, which and why?

**Project stakeholders and their values**

1. When (in time) do you expect your research to offer a concrete industrial application?
2. Who will have a role and be affected by this research/design project and its industrial application?
3. What do you think is important for them with this project, what do they/could they value from your project? Nature, society & the economy, the future?

**The designer’s view on this project and sustainability.**

1. Do you think “biobased R&D” benefits sustainability? Nature, society & the economy, future generations? How?
2. Do you think “biobased R&D” should benefit them? (Nature, society, future generations?)
3. Do you think this project benefits, or will contribute to benefit them? (Nature, society, future generations?)
4. Do you consider there is any specific challenge for your work/research with this project?

**The designer perspective with sustainability in technical design**

1. How have you been confronted in your work with sustainability aspects during the past 12 months, prior to this project?
   1. **If yes,** could you elaborate on the role of these aspects in your daily work?
   2. **If not,** what is your motivation for choosing this project over others? / participating on the competition?
2. Does your own personal opinion on sustainability issues play in determining the future direction of your work/research? How?
3. Would it be ‘‘good’’ for the quality of “bio-based” R&D to increase attention on sustainability issues, implications? How?
4. Do you think it would be beneficial for society if “bio-based” R&D would take into account sustainability issues? How?

**Expectations**

1. What are your expectations for the next months with this project and these sessions? What do you expect from my participation?
2. Do you consider these sessions part of your project, necessary, or as something extra to your work?
3. Do you have any further questions, or are there issues that have not been addressed?

## Design Space Workshops

As supplementary material from the design space workshops, we include two photographs of the white boards used during the first and third workshops (Figure SI-1 and SI-2 respectively). These board images contain some of the topics discussed during these sessions and how they were being discussed at the moment: Figure SI-1 is related to the production chain and life cycle of bioplastics, and different stakeholders involved; Figure SI-2 lists some of the sustainability aspects identified from the different investigations about, and with, the identified stakeholders.


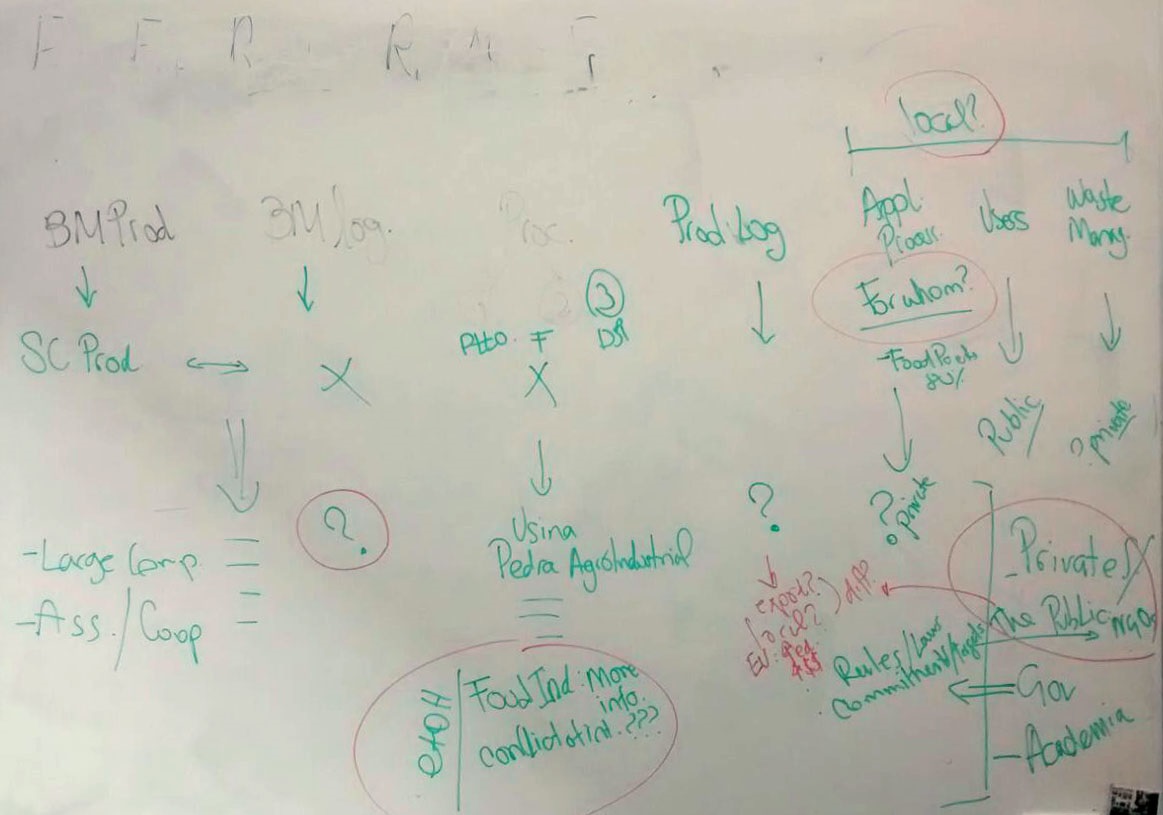


**C**

**B**

**D**

**A**

**Figure SI-1**. Photo of the board during the first workshop (see Table 1 in main text). Words above the dashed line (drawn on the photograph) indicate the generic production and life cycle stages of bioplastics, below it are different points discussed in relation to these stages. Letters A through D indicate these discussion points. A: extra activities in the production chain added by the team and related to the end-product application, product use, and waste management; B: location of users; C: role of biomass producers, and ethanol industries; D: application alternatives, prominently food packaging.

**
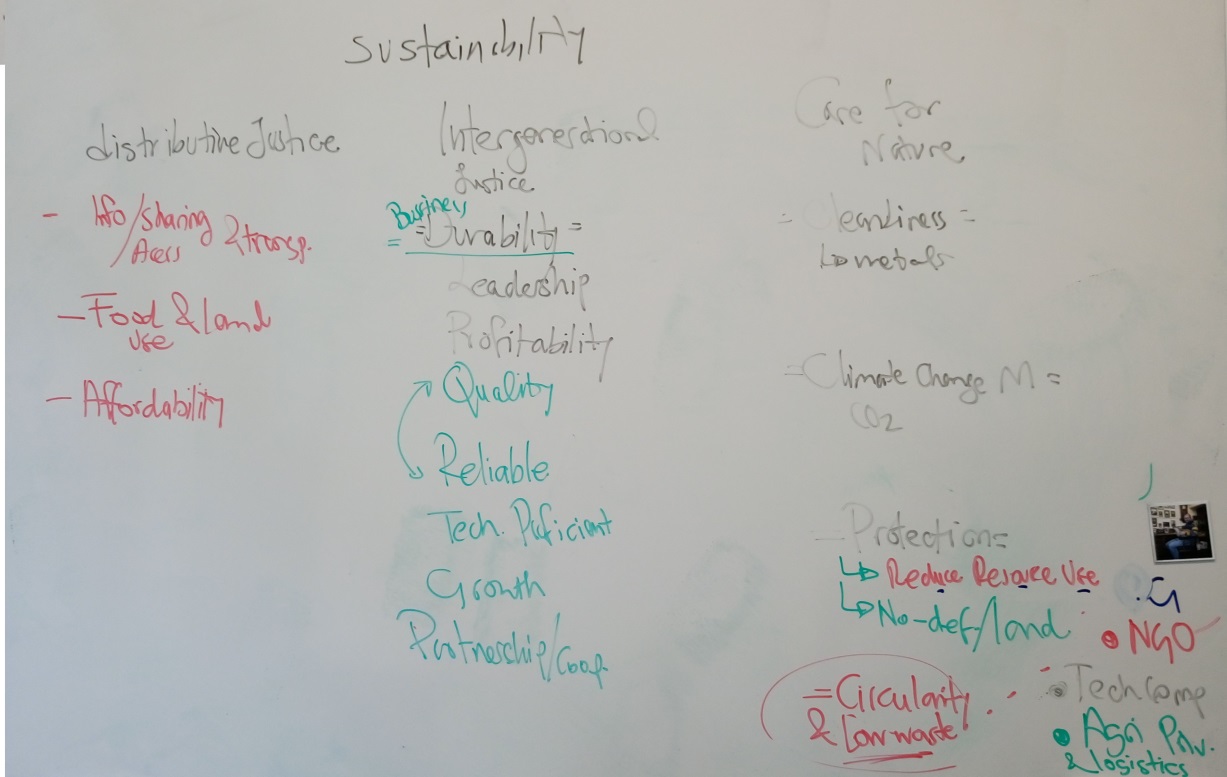
**

**Figure SI-2**. Photo of the board during the third workshop (see Table 1 in main text). In the bottom right corner are the stakeholder groups that were part of the discussion at that point: government (G), non-governmental organizations (NGO), technology companies (Tech Comp), and agricultural producers and biomass transportation stakeholders (Agri. Prdu. & logistics).

## Identified decision making processes

Summary of the different decision making processes identified along the development of the project with respect to the project variables: feedstock, products, process and business model.

| **Modulation** | **Alternatives and**  **decisions** | **Values** |
| --- | --- | --- |
| Feedstock - *Sucrose input* | | |
| De facto | Sugarcane juice | Designing feasibility |
| Reflective | The whole sugarcane can be processed and bagasse could be used in the process | Achievement, designing feasibility, entrepreneurship, food security, resource efficiency, process simplicity |
| Deliberate | Model the process parts for processing the whole crop as black boxes and add to the main conversion process |  |
| Final decision | Idem |  |
| Products - *Bagasse use and PHB form* | | |
| De facto | Bagasse as feedstock for energy production | Achievement, process simplicity, resource efficiency, technical feasibility |
|  | Pure BBP | Scientific focus |
| Reflective | Bagasse as 2G feedstock | Achievement, entrepreneurship, food security, resource efficiency |
|  | Co-polymers as main product | Achievement, entrepreneurship, product quality, resource efficiency |
| Deliberate | Investigate alternative uses for bagasse: 2G BBP production | Achievement, entrepreneurship, food security, product quality, resource efficiency |
|  | Investigate co-polymer compound alternatives and their production |  |
| Final decision | Model energy generation from bagasse and the production of pure BBP granules. The sustainability team suggested to include in the business plan a proposal to invest in researching 2G and wastewater BBP production | All of the above |
| Process *- Downstream Processing* | | |
| De facto | The use of solvents for PHB recovery is not desirable | Environmental safety |
| Reflective | There are other chemical, mechanical and enzymatic alternatives but they all carry disadvantage. The most common alternative is, however, based on solvents | Designing feasibility, entrepreneurship, environmental safety, product quality, technical feasibility |
| Deliberate | Model the process with an uncommon alternative that has low environmental impact. Use assumptions to cover missing data and estimate economic performance |  |
| Final decision | Idem. |  |
| De facto | Stand-alone plant that buys the sucrose feedstock |  |
| Reflective | The production of PHB can be integrated to an existing sugarcane mill. It would imply diverting some of the sugarcane juice for PHB production. In this way, streams can be integrated to recover energy/materials | Cooperation, entrepreneurship, resource efficiency |
| Deliberate | Model and compare the production process as integrated (witj sugar and ethanol production as black boxes) with as an independent process | Achievement, cooperation, entrepreneurship, resource efficiency, designing feasibility, process simplicity |
| Final decision | Idem. |  |
| Business Plan - *Business model* | | |
| De facto | The model has to be able to accommodate the integration of their process to an existing sugarcane mill, and a partnership with an existing sugarcane mill was the group’s initial idea for supporting this | Achievement, cooperation, entrepreneurship, resource efficiency |
| Reflective | The integrated process can be supported by other business models: BBP production as part of the same sugarcane company (merge model), or by licensing their patented technology to the mill companies (licensing model) | Cooperation, entrepreneurship, resource efficiency, leadership |
|  | If the business is integrated, there is the possibility to vary the production of any of the products as desired | Entrepreneurship |
|  | A licensing model implies confidentiality until a patent application is made | Scientific openness |
|  | A licensing model can be combined with a biodegradation step | Biodegradation |
|  | The merge model means losing ownership of the project | Ownership |
| Deliberate | Discard the licensing alternative and investigate further about the stand alone and partnership business models | Entrepreneurship, scientific openness |
| Final decision | Partnership model for the business | All of the above |
| Business Plan - *Target clients* | | |
| De facto | Clients are based on application and location. | Entrepreneurship |
| Reflective | The end user can have a prominent position to make sure the material gets degraded | Biodegradation |
|  | Biodegradability does not mean biodegradation | Biodegradation |
|  | There is a need for information about the biodegradability of the final product | Biodegradation |
|  | Clients that cannot or do not recycle plastic are potential clients for biodegradable plastics, others might just be as interested | Biodegradation, environmental safety |
|  | By targeting such clients the mixing of recyclable plastics with biodegradable plastics can be avoided | Biodegradation, environmental safety, renewability |
|  | Targeting a limited amount of clients can limit the business | Entrepreneurship. |
| Deliberate | Focus on clients that are interested or that could make sure the material is biodegraded | Biodegradation |
| Final decision | The business plan is focused on possible clients that can process the bioplastic | Biodegradation |
